# Subjective Visual Vertical and Postural Capability in Children Born Prematurely

**DOI:** 10.1371/journal.pone.0121616

**Published:** 2015-03-19

**Authors:** Maria Pia Bucci, Sylvette Wiener-Vacher, Clémence Trousson, Olivier Baud, Valerie Biran

**Affiliations:** 1 UMR1141 INSERM-Université Paris 7, Robert Debré Hospital, 48 boulevard Sérurier, 75019, Paris, France; 2 Vestibular and Oculomotor Evaluation Unit (EFEE), ENT Dept., Robert Debré Paediatric Hospital, 48 boulevard Sérurier, 75019, Paris, France; 3 Neuropsychologie, DHU PROTECT, Robert Debré Hospital, 48 boulevard Sérurier, 75019, Paris, France; 4 Neonatal Intensive Care Unit, Robert Debré Hospital, 48 boulevard Sérurier, 75019, Paris, France; Lyon Neuroscience Research Center, FRANCE

## Abstract

**Purpose:**

We compared postural stability and subjective visual vertical performance in a group of very preterm-born children aged 3-4 years and in a group of age-matched full-term children.

**Materials and Methods:**

A platform (from TechnoConcept) was used to measure postural control in children. Perception of subjective visual vertical was also recorded with posture while the child had to adjust the vertical in the dark or with visual perturbation. Two other conditions (control conditions) were also recorded while the child was on the platform: for a fixation of the vertical bar, and in eyes closed condition.

**Results:**

Postural performance was poor in preterm-born children compared to that of age-matched full-term children: the surface area, the length in medio-lateral direction and the mean speed of the center of pressure (CoP) were significantly larger in the preterm-born children group (p < 0.04, p < 0.01, and p < 0.04, respectively). Dual task in both groups of children significantly affected postural control. The subjective visual vertical (SVV) values were more variable and less precise in preterm-born children.

**Discussion-Conclusions:**

We suggest that poor postural control as well as perception of verticality observed in preterm-born children could be due to immaturity of the cortical processes involved in the motor control and in the treatment of perception and orientation of verticality.

## Introduction

Approximately 7% of premature children are born in France every year. These children have a greater risk of developing deficiencies and motor and cognitive impairments during childhood and adolescence [1]. Scott et al. [2] also reported increased rates of disorders in attention in a large population of preterm and extremely low birth weight children. In a recent review based on the meta-analysis of previous literature [3] researchers showed that very preterm-born children had deficits in visual-spatial abilities; the impairment in visual-motor integration was found to be more important in boys compared to girls. These authors advanced the hypothesis that the observed visual-spatial and visual-motor integration deficits could be due to the affected occipital-parietal-frontal neural circuitries in this children population.

In static conditions, postural control implies body orientation that is generally aligned to the gravity vector. It is well established that visual, vestibular and somatosensory information are important to control postural stability [4]. Children, like adults, use all these information to maintain their upright posture. Several studies on postural development reported age-related changes in the use of vision to control posture in infants [5–8] and in children [9,10]. All these studies are in agreement with the fact that young children are more visuo-dependent than adult subjects [11,12]. Lee and Aronson [8] first showed the role of visual information for a good control of posture in infants by studying the standing posture of seven infants from 13 to 16 months with optic flow pattern stimuli. These authors reported backward or forward body sways of infants, suggesting compensatory adjustments to posture made in response to the visual proprioceptive information they received. They also found that in the majority of cases, infants produced a sway in the same direction as that of the optic flow, to compensate adjustments of posture according to their visual information. Lee and Aronson concluded that to control postural stability, infants used more “visual proprioception” than “mechanical proprioception” information. In other words, visual inputs would be used more than other inputs to control posture. Several studies have been conducted to explore postural capabilities in young children. For instance, Forssberg & Nashner [13] reported that at the age of 4 it is still difficult to remain stable in an upright position with eyes closed, and at this age children are also less capable of solving a conflict of sensory disturbances [11,13,14]. Only after 7 years do children become able to compensate to a tendinous vibration [15]. Bair et al. [16] examined how children from 4 to 10 years can develop multisensory reweighing abilities to maintain a good postural control. These authors showed that the amount of reweighing increased with age, indicating the development of a better adaptive ability for postural control. Haas & Diener [17] showed that at age 4, children were capable of anticipating a disturbance, thus producing an anticipated postural adjustment, even if postural control is not mature yet. The production of such an adjustment requires the subject to know the geometry of the physical segments of the body and their orientation in space. It is well known that the body image builds itself on the basis of sensory information transmitted by the body during a movement and during interactions with the world that develops during childhood [18;19].

The perception of verticality is essential for life, particularly for bipedal orthostatic posture; the motor behavior organized around the vertical axes such as stabilization in space, body orientation, locomotion and spatial navigation depends on both perception and integration of verticality [20]. It is also well established that the perception of verticality requires a normal activity of the neural circuits centered on the superior parietal cortex [21–24], the insula [11,25] and the thalamus [26].

Wright et al. [27] explored both visual and vestibular contributions to vertical self motion perception in a small group of young adult subjects by using vertical linear oscillation and inertial visual scene motion. Interestingly these authors found that when visual and vestibular stimulation were combined, self-motion perception persisted even in the presence of visual-vestibular discrepancy and the visual input dominated the subjects self-motion, even if vestibular effects were also present. Bortolami et al. [28] suggested a model based on otolith activity for determining the spatial orientation of the verticality in humans by taking in account the gravitoinertial force. Note that vestibular inputs on the subjective visual vertical has an important role, as reported by Mazibrada et al. [29], and that such vestibular contributions are under adaptive processes [30].

While several studies deal with subjective visual vertical in adults, few focus on children. Our group [31] compared subjective visual vertical and postural capabilities in a group of children 6–8 years old and in a group of adult subjects. We showed that children were more unstable than adults during such dual-task and that they have less precise and less accurate representations of verticality than adults. This is probably due to the maturation of the cortical processes involved in the perception of verticality, which has not been attained in young children yet. Development of the central nervous system and visual-motor training contribute to the achievement of vertical evaluation and postural stability.

Several studies examined in children the effect of a secondary task on postural stability. Olivier et al. [32] advanced the hypothesis of a presence of two independent attentional mechanisms, one that is responsible of the balance control and the other to the secondary task; such two mechanisms could interfere depending on the difficulty of these two tasks. According to Huxold et al. [33] the complexity of a secondary task could affect in a different way postural stability by increasing or decreasing it, depending on its difficulty.

Based on all these findings, we made the hypothesis that very preterm-born children could have poor postural control compared to full-term children and that they could have more difficulty to focus attention on the secondary task leading to poor representation of verticality.

In the present study we compared postural capabilities and subjective visual vertical performance in a group of very preterm-born children of 3–4 years old versus a group of age-matched full-term children. We made the prediction that immaturity of the central nervous system as well as reduced attentional resources of very preterm-born children could lead to larger postural sway and imprecise visual vertical perception.

## Methods

### Subjects

Children born in 2010 at the Neonatal Intensive Care Unit of Robert Debré Hospital after 24 to 28 completed weeks of gestation were included in the present study. Follow-up involved cerebral magnetic resonance imaging (MRI) at term-equivalent age without sedation, ophthalmologic (visual acuity) and orthoptic examination (the absence of heterotropia) and audiometric test at 2, 12, 36 months, as well as medical and psychometric assessments up to the age of 7 years.

The developmental quotient (DQ) of 24-month preterm infants was determined by a specialized psychologist based on the revised Brunet–Lezine Test. It is an early childhood psychomotor test developed in France from 1943 and revised between 1994 and 1996 on a sample of 1032 French children [34] with rigorous methods including the evaluation of test–retest reliability and internal reliability, both of which were high [35]. The test is divided into four sections: posture, oculomotor coordination, socialization and language.

Brain MRI at term-equivalent age was used to evaluate the presence and degree of white matter disease, including gray matter injury (GMI) and white matter injury (WMI), and punctate white matter lesions. White matter and gray matter abnormalities in brain MRI were scored based on the system of Woodward et al. [36]. The WMI score was obtained by adding the subscores of white matter signal abnormality (the so-called diffuse excessive high signal intensity, DEHSI), periventricular white matter volume loss, presence of cystic abnormalities, ventricular dilatation, and thinning of corpus callosum. The GMI score was obtained by adding the subscores of cortical abnormalities, quality of gyral maturation, and size of subarachnoid space.

Children characteristics are described in Table 1. Twenty-two children born prematurely (gestational age from 25 to 27 weeks) and twenty-two full-term age-matched children participated to the study. ANOVA run on birth weight, gestational age, number of boys and girls, walking age and age at assessment for the two groups of children (preterm and full-term born children) showed significant difference on birth weight, gestational age and walking age (all p< 0.001). Audiometric test and ophthalmologic/orthoptic examinations were normal for all children; two preterm children only wore glasses (C11 myopia correction and C16 hypermetropia correction). The global developmental quotient (DQ) for the Brunet Lezine’s test was normal (value > of 70) for the majority of preterm children examined; three children only (C6, C13 and C15) showed a lower quotient, meaning that developmental abilities were not completely developed. Cerebral MRI was performed in 19 preterm children at term-equivalent age corrected; it was normal in 9 children only. The other preterm children had abnormal scores for white matter injury and white matter signal intensity.

The twenty-two full-term control children had normal values of ophthalmologic/orthoptic and audiometric examination. The age of children chosen in this study was based on preliminary experiments run on children of 2–4 years old: children of about 2 years old were not able to perform the two tasks correctly. Frequently, they were also afraid of the dark. Consequently we decided to test children > 3 years old.

**Table 1:** Clinical characteristics of children tested.

	Preterm n = 22	Controls n = 22	P values
Birth weight (gr)	863.6 [650–1130]	3670 [3300–3860]	0.001
Gestational age (weeks)	26.4 [25–27.6]	39.1 [38–40]	0.001
Boys/girls	16/6	15/7	
Walking age (months)	16.5 [13–21]	13.2 [12–16]	0.001
Normal MRI at 40 corrected GA	9/19	ND	
Age at assessment	3.8 [3.1–4.9]	3.9 [3.1–5]	

Mean and minimum and maximum values (in square brackets) of the birth weight (in gr), gestational age (in weeks), the number of boys and girls, the walking age (in months), the number of preterm children with normal MRI at 40 corrected GA and the age at assessment. p values are also shown for significant difference between the two groups of children.

Exclusion criteria were the presence of a genetically defined syndrome, severe congenital malformation adversely affecting life expectancy, or abnormality known to affect neurodevelopment.

The investigation adhered to the principles of the Declaration of Helsinki and was approved by our institutional Human Experimentation Committee (Comité Consultatif d’Ethique Local, Robert Debré Pediatric Hospital). Informed written consent was obtained from the children’s parents after an explanation of the experimental procedure.

### Experimental set up

The test took place in a dark room. Child was required to stand upright on the platform. A large black curtain was suspended from the ceiling to form a semi cylindrical black space around the children in order to avoid any visual references. The visual vertical perception was assessed with a homemade subjective visual vertical system composed of a phosphorescent tube and a fluorescent cardboard figure looking like a clown. This clown was placed two meters away from the child, at eye level, and the child could moved it to the left or to the right with a remote control.

### Experimental procedure

Tasks are similar to those used previously by our group [31] and are described below. Children were asked to stand upright on the platform and the position of the feet was as follows for all children: heels 4 cm apart and feet spread out symmetrically at a 30° angle with respect to the child’s sagittal axis. Arms were vertical along the body (note that this position is appropriate for subjects of all heights and it is given by the Association Française de Posturologie [37]. Children held the remote control with their hands in front of their belly buttons. The movement required by the control remote was just a contraction of the thumbs, and the keyboard was only 40 gr. On the keyboard, there were only two buttons: the right button turned the clown to the right, and the left button turned the clown to the left.

Experimental session included five conditions: OKN+SVV at 40°/s left and 40°/s right, No OKN + SVV, DARK+FIX and DARK+EC; for three of them only SVV measure was also recorded (OKN+SVV at 40°/s left and 40°/s right, No OKN + SVV). Below each condition we will describe.

In the conditions OKN+SVV 40°/s left and 40°/s right, an optokinetic stimulation was projected on the black curtains: 360 dots of 0.34 cm (0.235°) in diameter subtending 12° (~41.69 cm) of the visual field. The dots rotated to the right (clockwise) or to the left (counterclockwise) with constant angular velocity of 40°/s.

One more condition was performed in the dark without any optokinetic visual stimuli (condition No OKN + SVV).

For these three conditions, SVV measurement was also recorded. Prior to each trial, the experimenter inclined the clown on the right or on the left side randomly, at different angle of tilt. The child then had to straighten the clown up until it reached verticality. At the same time postural stability was recorded. Before starting the experiment, we trained children on the DARK+SVV condition, without recording it, until we were sure that children had understood the task and performed it well.

For the three conditions OKN+SVV at 40°/s left and 40°/s right and No OKN + SVV three trials were run.

Two control conditions were also run to assess postural stability without SVV measurement: one condition (DARK+FIX) where the child was asked to fixate the clown that was vertically (0°) aligned; and another condition (DARK+EC) where both eyes were closed by a patch while the child was asked to mentally recall the image of the clown. For each of these conditions two trials were run.

### Data acquisition

A platform (principle of strain gauge) consisting of two dynamometric clogs (standards by Association Française de Posturologie, produced by TechnoConcept, Céreste, France) was used to measure postural stability. The excursions of the center of pressure (CoP) were measured for 12.8 seconds and the surface of the CoP was calculated following the standards proposed by Gagey & Weber [38]; the equipment included a 16-bit analog-digital converter. The sampling frequency of the CoP was 40 Hz.

### Postural parameters

We analyzed the surface area, the length in the medio-lateral direction and the mean speed of the center of pressure (CoP). The surface area and the length allowed for the efficient measurement of CoP spatial variability; the surface area of the CoP corresponds to an ellipse with 90% of CoP excursions [39]. The length of CoP is the path of the center of pressure in the medio-lateral direction. This direction was chosen because the optokinetic stimulation could affect postural stability more in the medio-lateral than in the anterio-posterior axis [31]. The mean speed of the CoP represents a good index of the amount of neuromuscular activity required to regulate postural control [40,41].

### SVV evaluation

We examined the SVV value that was, in degrees, the error from the absolute visual vertical at zero degree. For each child we calculated the mean of the three responses (in absolute value) that were performed for the three conditions (OKN+SVV at 40°/s left and 40°/s right respectively and No OKN + SVV). We decided to record three times only the SVV in order to avoid fatigue.

### Statistical analysis

We performed an ANOVA using the aforementioned conditions as main factor and the two groups of children (preterm and full-term born children) as inter-subjects factor for all postural parameters (surface, length in medio-lateral direction and mean speed of the CoP).

An ANOVA test was also performed on the mean SVV absolute values. The post-hoc comparisons were made with the least significant difference (LSD) test. The effect of a factor was considered significant when the p-value was below 0.05.

## Results

**Fig 1:**
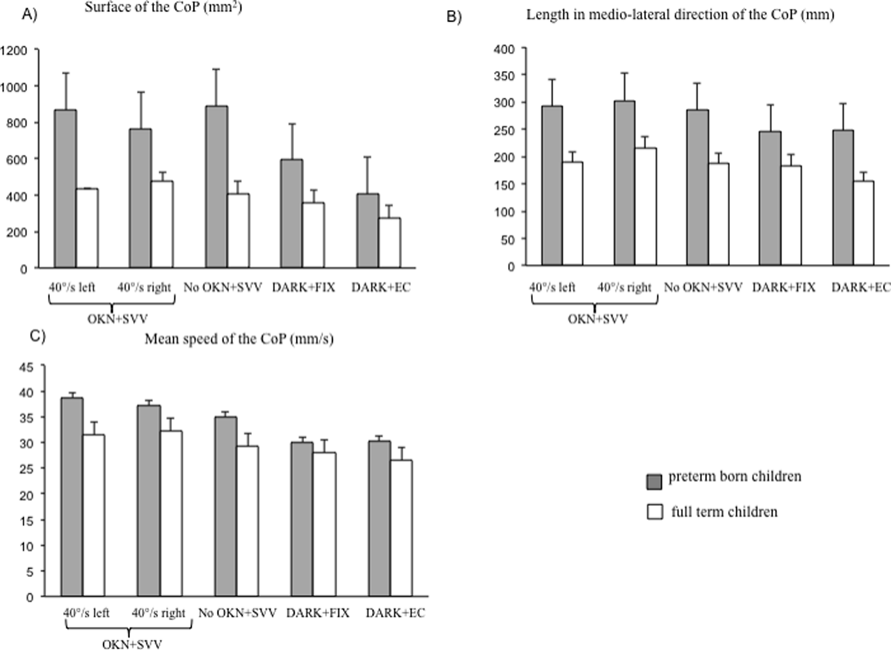
Postural parameters for the different conditions. Mean of Surface of the CoP (A), of length of CoP in the medio-lateral axis (B), and of mean speed of CoP (C) for the five different conditions (OKN+SVV: 40°/s left and 40°/s right, respectively, No OKN+SVV, DARK+FIX and DARK+EC) for the two groups of children tested. Verticals bars indicate the standard error. Asterisks indicate that the value is significantly different at the post-hoc test (p < 0.05).

Fig. 1A shows the surface area of CoP for five different conditions for both groups of children tested (preterm and full-term born children). The ANOVA showed a significant effect of group (F_(1,42)_ = 4.22, p < 0.04); pre-term born children showed significantly larger surface of the CoP than full-term children. ANOVA also showed a significant effect of the condition (F_(4,168)_ = 5.86, p > 0.0002). The post-hoc test showed that the surface of the CoP was significantly smaller in the fixation and eyes closed conditions (DARK+FIX and DARK+EC) with respect to the other conditions (40°/s left and 40°/s right and No OKN + SVV, all p > 0.01).

Fig. 1B shows the length of CoP in the medio-lateral direction for the five different conditions for both groups of children tested (preterm and full-term born children). ANOVA showed a significant effect of group (F_(1,42)_ = 6.01, p < 0.01): pre-term born children had significantly longer length of the CoP than full-term children. ANOVA also showed a significant effect of condition (F_(4,168)_ = 4.45, p<0.001). The post-hoc test showed that the length of the CoP was significantly shorter in the fixation and eyes closed conditions (DARK+FIX and DARK+EC) with respect to the optokinetic conditions (40°/s left and 40°/s respectively, both p > 0.01); furthermore, the length of CoP recorded in the No OKN + SVV was significantly shorter than in the optokinetic condition (40°/s right, p < 0.04).

Fig. 1C shows the mean speed of CoP for the five different conditions for both groups of children tested (preterm and full-term born children). ANOVA showed a significant effect of group (F_(1,42)_ = 4.40, p < 0.04): the mean speed of the CoP was significantly higher in pre-term born children compared to that of full-term children. ANOVA also showed a significant effect of condition (F_(4,168)_ = 4.78, p<0.001). The post-hoc test showed that the mean speed of the CoP was significantly smaller in the fixation condition with respect the other SVV conditions (40°/s left and 40°/s right and No OKN + SVV, all p > 0.04); the mean speed of the CoP in the eyes closed condition was also significantly smaller than in the two optokinetic conditions (40°/s left and 40°/s right, both p > 0.006).

**Fig 2:**
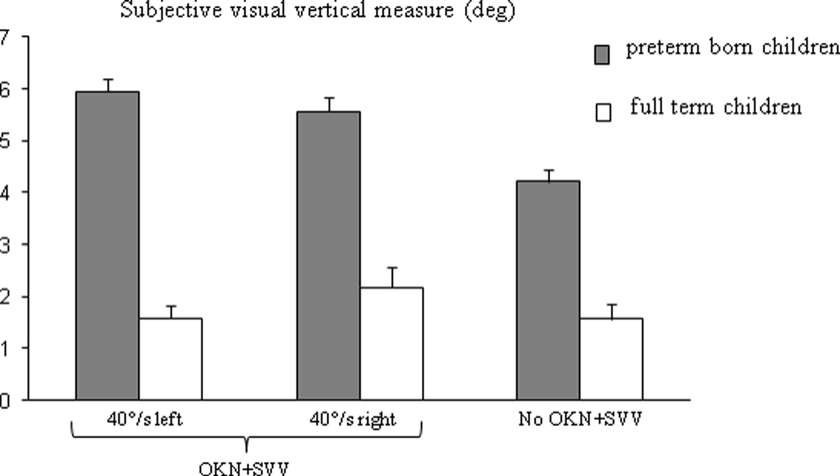
Mean Subjective visual vertical measure. Mean values of the SVV for the three different conditions (OKN+SVV at 40°/s toward the left and the right and the condition No OKN+SVV) for the two groups of children tested. Verticals bars indicate the standard error.

Fig. 2 shows the subjective visual vertical measures in absolute values for the three conditions (the OKN+SVV: 40°/s left and right direction and the No OKN+SVV condition) for both groups of children tested (preterm and full-term born children, respectively). ANOVA showed a significant effect of group only (F_(1,42)_ = 43.53, p < 0.001). The subjective visual vertical values for pre-term born children were significantly larger than those reported in full-term born children. The post-hoc test failed to show significant differences in the three conditions.

Finally we explored whether we could find a different postural control between preterm-born children with normal and abnormal MRI. As shown in Table 2, ten preterm children had abnormal MRI results (and two of them showed also abnormal Brunet Lezine’s score) while only nine preterm children had normal MRI (three children had no MRI done). Because of age difference between these two sub-groups and also due to the limited number of children in each group only a qualitative analysis can be done. Table 3 shows the data for the surface of the CoP: for all conditions tested, the group of preterm children with abnormal MRI results has larger value of the surface of the CoP. Similarly, larger values have been also observed for the other postural parameters measured.

**Table 2:** Clinical test and MRI results in preterm children.

Child	Walking date (months)	Postural DQ	Oculomotor coordination DQ	Language DQ	Social DQ	Global DQ	Writing hand	MRI
C1	17	95	79	87	107	90	R	1
C2	15	ND	ND	ND	ND	ND	R	1
C3	19	78	83	74	94	80	R	1
C4	17	107	90	71	95	87	R	1
C5	14	85	83	81	105	86	R	ND
C6	21	**67**	**51**	**61**	**56**	**57**	R	1
C7	16	ND	ND	ND	ND	ND	R	1
C8	15	ND	ND	ND	ND	ND	L	0
C9	11	91	81	68	103	82	R	ND
C10	19	88	79	63	74	75	R	0
C11	17	94	85	94	85	91	L	0
C12	18	85	82	88	104	88	L	1
C13	18	**55**	**67**	**51**	**67**	**61**	R	1
C14	16	109	72	76	108	86	R	ND
C15	17	81	70	71	73	74	ND	0
C16	18	82	74	72	70	71	R	0
C17	15	84	101	76	78	85	R	0
C18	13	84	90	65	84	81	R	1
C19	13	89	86	73	97	83	L	0
C20	15	90	87	74	76	81	ND	0
C21	21	102	**65**	72	72	75	R	1
C22	18	103	99	73	92	89	R	0

Brunet Lezine’s test results: developmental quotient (DQ) for postural capability, oculomotor coordination, language and social capabilities; the global quotient is also reported. The bold values are abnormal. Writing hand left (L) or right (R) tested the day of experiment. ND test not done. Magnetic resonance imaging MRI: normal (0); abnormal (1).

**Table 3:** Mean surface area of the CoP for the preterm born children with abnormal and normal MRI results.

Preterm children	40°/s left	40°/s right	No OKN+SVV	DARK+FIX	DARK+EC
**MRI abnormal** (n = 10)	1307 ± 443	876 ± 207	1178 ± 290	688 ± 188	483 ± 107
**MRI normal** (n = 9)	657 ± 229	657 ± 229	794 ± 260	669 ± 170	370 ± 71

Mean of Surface of the CoP and the standard error for the five different conditions (OKN+SVV: 40°/s left and 40°/s right, respectively, No OKN+SVV, DARK+FIX and DARK+EC) for the two groups of preterm born children tested with abnormal (10 children) and normal (9 children) MRI results.

## Discussion

The main findings of this study are as follows: (i) Postural performance is different in preterm-born children from that of age-matched full-term children; (ii) Dual task in young children affects postural control; (iii) SVV values are more variable and less precise in preterm-born children. These findings are discussed individually below.

### (i) Postural performance is different in preterm-born children

The new finding of this study is that postural performance is different in preterm born children from that of age-matched full-term children. Indeed all postural parameters tested in all conditions run (with and without vision stimulation) are larger in these children. This result is in line with the existing studies already cited in the Introduction showing that postural stability is poor in young children and improves with age; indeed the surface area and the mean speed of the CoP are larger in young children [42,43]. This study also provides objective data on motor impairment in preterm-born children that confirm what has been reported by several authors by subjective tests only [3]. According to the study of Milner and Goodale [44] that pointed out the role of the dorsal visual stream in the control of visual-motor capabilities, visual-motor integration deficits reported in preterm-born children by our and previous other studies could be due to impaired functioning of the occipital-parietal-prefrontal cortex, involved in visual-motor control. Moreover, recall that MRI findings showed white matter injury and white matter signal intensity in 10 preterm children. It is well known that such diffuse excessive high signal intensity (DEHSI) and punctate white matter (WM) lesions have been assumed to represent a diffuse and subtle form of WM injury in preterm children [45,46]. Also diffuse WM injury is generally held responsible for the high incidences of mental and behavioral disorders in very preterm-born infants, and the presence of punctuate WM lesions is known to be associated with alterations in later neurodevelopment [46,47]. Consequently the poor postural control qualitatively reported in these 10 preterm children with respect to the group of 9 preterm children with normal MRI is in line with our hypothesis on the immaturity of cortical functions responsible for postural control. This issue needs to be further explored by a study combining MRI and behavioral experiments in a larger number of preterm children.

### (ii) Dual task in young children affects postural control

The effect of a secondary task on postural stability is similar in both groups of children for all postural parameters examined. The present study shows that the DARK+FIX and the DARK+EC condition were the conditions where children showed smaller surface, length and mean velocity of the CoP compared to the conditions where children had to do the secondary task (vertical visual subjective evaluation) at the same time, independently of the presence or not of the optokinetic visual stimulation (OKN+SVV and No OKN+SVV condition). This result is new and suggests that the attention allowed in dual-task is similar in preterm-born children to that of control children. Recall that the secondary task used in the present study is to perceive and appreciate subjective visual vertical, which is a cognitive task. Studies dealing with effect of dual-task on postural stability in children from our group [43,48,49] showed a change in the postural parameters (large surface, length and mean velocity of the CoP) most likely due to the fact that children had to shift their attention toward the secondary task, leading to worse postural control. Such behavior is in line with the U-shaped non-linear interaction model of Huxhold et al. [33], who suggested that when the attentional demand for the cognitive task increases (*i*.*e*., subjective visual vertical task), a degradation of the postural sway is observed.

Finally, another result has to be pointed out from this study: the role of vision on postural control in children 3–4 years old. We showed that postural values were similar for both groups of children for the two conditions (with and without vision, respectively DARK+FIX and DARK+EC condition) in contrast to the previous work of Shumway-Cook et Woollacott [11] reporting that children of 4–6 years old were more visuo-dependent than older children (7 years old). Most likely children shift their attention in order to reduce as much as possible their postural sway during closed eyes condition. Note, however, that the role of vision on postural stability needs further examination particularly by testing simultaneously how visual and other sensory inputs could affect posture in children.

### (iii) Vertical visual subjective is more variable and less precise in preterm-born children

This study shows that the values of subjective visual vertical in preterm-born children were more variable and less accurate than those reported in age-matched full-term children, and that such error was independent from the presence or not of optokinetic stimulation. We could make the hypothesis that preterm children showed poor estimation of the subjective visual vertical because of their later cortical maturation of structures permitting a precise enough analysis of each piece of the multisensory referent information to construct their visual vertical. This hypothesis is in line with the EEG study of Lopez et al. [50] examining the subjective visual vertical in a group of 12 adult subjects. These authors reported that SVV involves large cortical processing with an early activation (75–105 ms) located in the right lateral temporo-occipital cortical areas and a later activation (260–290 ms) of bilateral temporo-occipital and parieto-occipital cortical areas. The early activation could involve lateral and ventral visual pathways in relation with the treatment of perception and orientation, while the later activation would involve multisensorial processes, as information on gravity and on the body position using vestibular, muscular and proprioceptive inputs. Such central structures could be still immature in preterm-born children, leading to poor SVV estimation.

## Conclusion

The present study shows that postural control and SVV evaluation is impaired in preterm-born children (3–4 years of age) with respect to those of age-matched full-term children. We suggest that such difference could be due to immaturity of the cortical processes involved in the motor control as well as in the perception of verticality. In order to find whether visual experience during day life together with development of the central nervous system could improve such capabilities, further longitudinal studies are needed on a larger population of this kind of children.
